# Impact of a Novel Social Work Program on Access to Tertiary Care

**DOI:** 10.5334/aogh.3585

**Published:** 2022-04-06

**Authors:** Athanasios Burlotos, Paola Alejandra Vargas Díaz, Manuel Alejandro Hernández Pacheco, Lorena Daniela Ponce de León Angel, Miriam Morales Camas, Jesús Sepulveda-Delgado, José Manuel Pérez-Tirado, Santiago Ortiz-Barragan, Anthony T. Fuller, Gustavo Nigenda

**Affiliations:** 1 Duke University Global Health Institute, Durham, North Carolina, US; 2 Duke University Medical School, Durham, North Carolina, US; 3 Compañeros en Salud, Jaltenango de la Paz, Chiapas, MX; 4 Instituto Nacional de Pediatría, Ciudad de México, MX; 5 Hospital Regional de Alta Especialidad Ciudad Salud, Tapachula, Chiapas, MX; 6 La Universidad Nacional Autónoma de México, Ciudad de México, MX

## Abstract

**Background:**

In the movement for global health equity, increased research and funding have not yet addressed a shortage of evidence on effectively implementing context-specific interventions; one unmet need is facilitating access to specialty care within the public health sector in Mexico. Compañeros en Salud has been piloting a novel program, called Right to Healthcare (RTHC), to increase access to specialty care for the rural poor in Chiapas, Mexico. The RTHC program incorporates social work, patient navigation, referrals, direct economic support, and accompaniment for patients.

**Objectives:**

This study evaluates the effectiveness of the RTHC program. Primary outcomes analyzed included acceptance of any referral and attendance of any appointment. Secondary outcomes included acceptance of the first referral and rate of appointment attendance for patients with an accepted referral.

**Methods:**

Using referral process data for the years 2014 to 2019 from a public tertiary care hospital in Chiapas, 91 RTHC patients were matched using 2:1 optimal pair matching with a control cohort balancing covariates of patient age, sex, specialty referred to, level of referring hospital, and municipality.

**Findings:**

RTHC patients were more likely to have had an accepted referral (OR 17.42, 95% CI 3.68 to 414.16) and to have attended an appointment (OR 5.49, 95% CI 2.93 to 11.60) compared to the matched control group. RTHC patients were also more likely to have had their first referral accepted (OR 2.78, 95% CI 1.29 to 6.73). Among patients with an accepted referral, RTHC patients were more likely to have attended an appointment (OR 3.86, 95% CI 1.90 to 8.57).

**Conclusions:**

The results demonstrate that the RTHC model is successful in increasing access to specialty care by both increasing referral acceptance and appointment attendance.

## Introduction

### Background

In 2015, the United Nations debuted the sustainable development goals (SDGs). The third goal addresses health and well-being across all ages [1]. While progress has been made, it has become clear that the methods used to drive initial improvements in the global burden of disease (GBD) will not be sufficient to meet the pace outlined by the SDGs. Notably, the GBD 2017 SDG Collaborators highlight that curative interventions which drove initial progress will need to be replaced by “prevention-oriented policy action and investments to achieve SDG aims [2].” This will require investing in and strengthening existing health systems. While an increase in research has led to documentation of global health inequity and the need for action, a paucity of implementation research, especially context-specific implementation research, hinders advancement towards global health equity [3]. The increased global health research output has not yet offered sufficient evidence on how to implement global health interventions, for which increasing funding has become available [3].

Mexico, despite being the world’s 15^th^ ranked economy by GDP with rapid growth [4,5], endures increasingly high levels of inequality [6]. This is demonstrated by its GINI coefficient of 0.45, classifying Mexico as a high inequality country [7]. The resulting poverty is geographically distributed and disproportionately affects the indigenous population, exemplified by the overlap between the poorest states and the states with the highest portion of indigenous language speakers [8,9]. Based on GDP per capita, Chiapas is both the poorest state and the most indigenous [8,9]. The average municipal Human Development Index in Chiapas is 0.664, well below the national average of 0.762 [10].

### The Mexican Healthcare System

Health service delivery and financing in Mexico can be grouped into three categories of payer and provider–social insurance, the public sector, and the private sector. The integrated payment-provider social insurance schemes are coupled to employment status. These programs cover formal sector workers and include programs such as IMSS (formal private sector workers, 33% of the population [11 (p17)]) and ISSSTE (federal government employees, 7% of the population [11(p17)]). There are additional social insurance schemes for state government employees, military members, and PEMEX employees. Each insurance scheme is a separate financial entity and utilizes a separate vertical network of providers and hospitals. The public sector is funded through federal and state general tax revenue and manages its own vertical network of healthcare infrastructure. The public sector provides a limited benefits package to those without social insurance and is discussed in more detail below. Lastly, the private sector provides services on an out-of-pocket fee-for-service basis. While only a minority privately-insured population relies entirely on this sector for outpatient and inpatient care, the private sector is often utilized due to barriers in access or gaps in service availability in the public sector [12].

### The Public Sector in Mexico

Mexico aims to provide universal healthcare coverage through the public sector. This began with the establishment of the Social Protection for Health System and its financial and operational branch “Seguro Popular” launched in 2004 in response to financial inequity within the healthcare system in Mexico. Seguro Popular was the first push towards universal health coverage in Mexico and provided services to a vast portion of the population [13]. The program had 53.5 million enrolled citizens in December of 2018, representing 44.7% of the population [14,15]. Although Seguro Popular was officially canceled in 2018, universal healthcare remains the stated goal as the public sector undergoes reform [16]. (Due to a lag in implementation, the authors do not believe that the healthcare reforms significantly affected the study population during the study period.) The segmented nature of the Mexican health system leads to an unequal distribution of availability and quality of services. This is most obvious for patients which rely entirely on the public sector, whose coverage is limited compared to the social insurance schemes. For example, although Seguro Popular financed a total of 56 high-cost hospital interventions, it did not cover treatment for myocardial infarctions over the age of 65, or chronic dialysis [13]. Within Mexico, healthcare resources, which are in total lower than OECD averages, [11 (p xxvi)], are especially limited in rural areas. This is due to a highly centralized healthcare infrastructure, with only 3.3% of hospitals located in rural areas [11 (p102)].

Within Chiapas these inequalities compound for the close to 75% of the population which relies on the Public Sector for healthcare [17], and are especially limiting for access to specialty care. A useful metric is the percent of the population registered with the Ministry of Health or other health insurance scheme, which until recent reforms was required to access specialty care. Although through 2018 proof of registration with the Ministry of Health was a stated requirement to access all healthcare levels, in practice lack of registration would rarely prevent a patient from accessing primary care level services; however, registration with the Ministry of Health or other payer was a strict requirement to access secondary or tertiary care. Therefore, the percent of the population unregistered with any healthcare service, a government indicator, is a useful metric to estimate the population without access to specialty care. In 2020, the percentage of the population not registered with any healthcare service was 26.5% nationally [15], and 33.3% in the state of Chiapas [17]. For the rural population, access to specialty care is further restricted by limited hospital personnel, poor civil and hospital infrastructure, large geographic distances, and limited availability of medical supplies. Furthermore, the same lack of formal economy that drives the rural poor to rely on public sector services (as they are largely excluded from jobs which are associated with social insurance schemes), predisposes them to the economic conditions which make them least prepared to overcome these barriers.

### Compañeros en Salud and the Right to Health Care Program

The lack of availability of health services for patients in Chiapas led the international NGO Partners in Health to collaborate with the local Ministry of Health via its affiliate, Compañeros en Salud, in the Frailesca and Sierra Madre regions of Chiapas in 2011 [18]. Since then, Compañeros en Salud has been able to staff and support 11 vacant rural healthcare clinics, increasing access to primary care in the region [19]. Despite this increase in primary care access, significant, often prohibitive, barriers still exist for patients in the region when seeking access to specialty care. These factors include the intersection of economic inequality, geographic factors, limited civil and healthcare infrastructure, social and cultural discrimination, and difficulty navigating a highly complex health system. Additionally, the indigenous population in Mexico, which is prominent in the study region, faces additional barriers such as a lack of translators of indigenous languages, and further geographic isolation [20]. To help their rural patients access specialty care, Compañeros en Salud implemented a free, comprehensive support system known as the Right to Health Care (RTHC) program. The RTHC program addresses barriers faced by rural patients within Compañeros en Salud’s primary care network when accessing the secondary and tertiary care services in the public sector to which all Mexican citizens are entitled. Given how the barriers in the region reflect the barriers faced by patients in Mexico’s public health sector more broadly, the Frailesca and Sierra Madre regions of Chiapas are well-suited to study this new model of increasing access to specialty care through bolstering and supporting the existing public sector.

The RTHC provides interventions to help patients overcome socioeconomic barriers to healthcare, and directly address gaps in their public sector benefits. The program is innovative and unique in that it comprehensively addresses barriers to healthcare by funding patients’ medical and non-medical costs, coordinating social work services, and providing patient navigation support and accompaniment. The RTHC program primarily utilizes patients’ existing public sector benefits, but also provides funding to obtain healthcare services when gaps in availability or coverage make the service unavailable in the public sector [21]. Furthermore, the RTHC program is unique in that it is an integrated social work and patient navigation program located at the primary level of care in a rural area. This contrasts with other social work departments in Mexico, which are in larger urban hospitals. A recently published theoretical economic model of a subset of RTHC program patients predicts a significant impact and cost-effectiveness, with an average of 14.8 Quality-Adjusted Life Years (QALYs) added for included patients, at a cost per QALY of $388 USD [22]. This promising preliminary analysis, the uniqueness of the RTHC program model, and the paucity of evidence in the global health literature on effective interventions to increase access to specialty care while working within existing health systems, all demonstrate the need for an empirical outcome analysis of the RTHC program.

### Objectives

**Figure 1:**
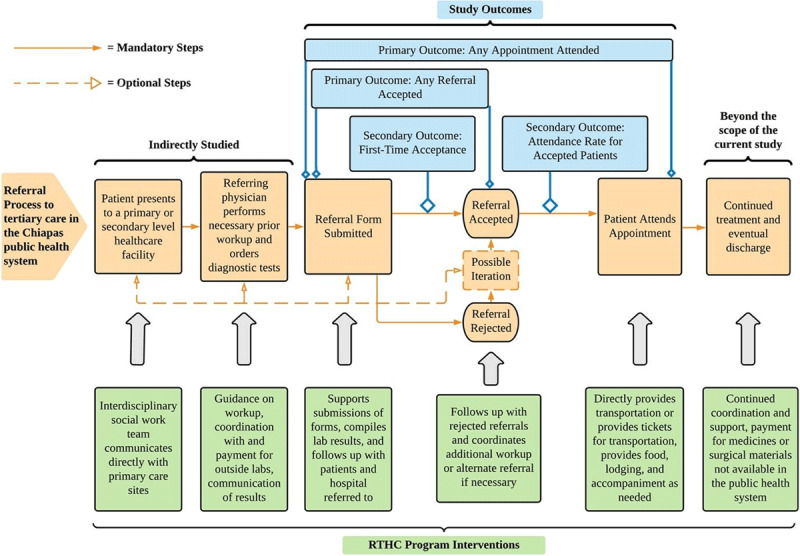
Summary of the relationships between the referral process, RTHC program interventions, and study outcomes.

By evaluating the implementation of a novel social work program, the study aims to evaluate the effectiveness of this new model in increasing access to specialty care in the context of rural Chiapas. The relationship between the referral process in Chiapas, interventions of the RTHC program, and the outcomes of the study are detailed below in ***Figure 1***.

## Methods

### Ethics

IRB approval was received from Duke University (Number: 2021-0231). Additionally, bioethics approval was obtained from the State of Chiapas Secretary of Health (signed by MTRA. Marianna Lazos Salgado). Lastly, approval was granted by the Research Committee at Hospital Regional de Alta Especialidad “Ciudad Salud” (Memo Number: HRAECS-DGA/JDID/CI/004/2021).

### Setting

Compañeros en Salud operates 11 primary care clinics in the rural, mountainous Frailesca and Sierra Madre regions in Chiapas, Mexico. These clinics serve a catchment area of 122 469 thousand people, roughly centering around the town and municipal center of Jaltenango de la Paz, where Compañeros en Salud is based. Within this region there is one secondary level hospital; however, due to infrastructure and staffing difficulties the hospital frequently is unable to provide standard secondary level care. There are no tertiary level care facilities in the region.

The Hospital Regional de Alta Especialidad “Ciudad Salud” (henceforth “Hospital Ciudad Salud”) is a public federal tertiary care facility located in Tapachula, the second-largest city in Chiapas. Hospital Ciudad Salud is the only public sector tertiary care facility in Chiapas for adults and serves patients from throughout the state and neighboring states. Due to geography and infrastructure, a one-way trip to Hospital Ciudad Salud from the communities which Compañeros en Salud serves takes 12+ hours by private vehicle, and between 1 and 3 days using public transportation.

### Data Collection

**Figure 2:**
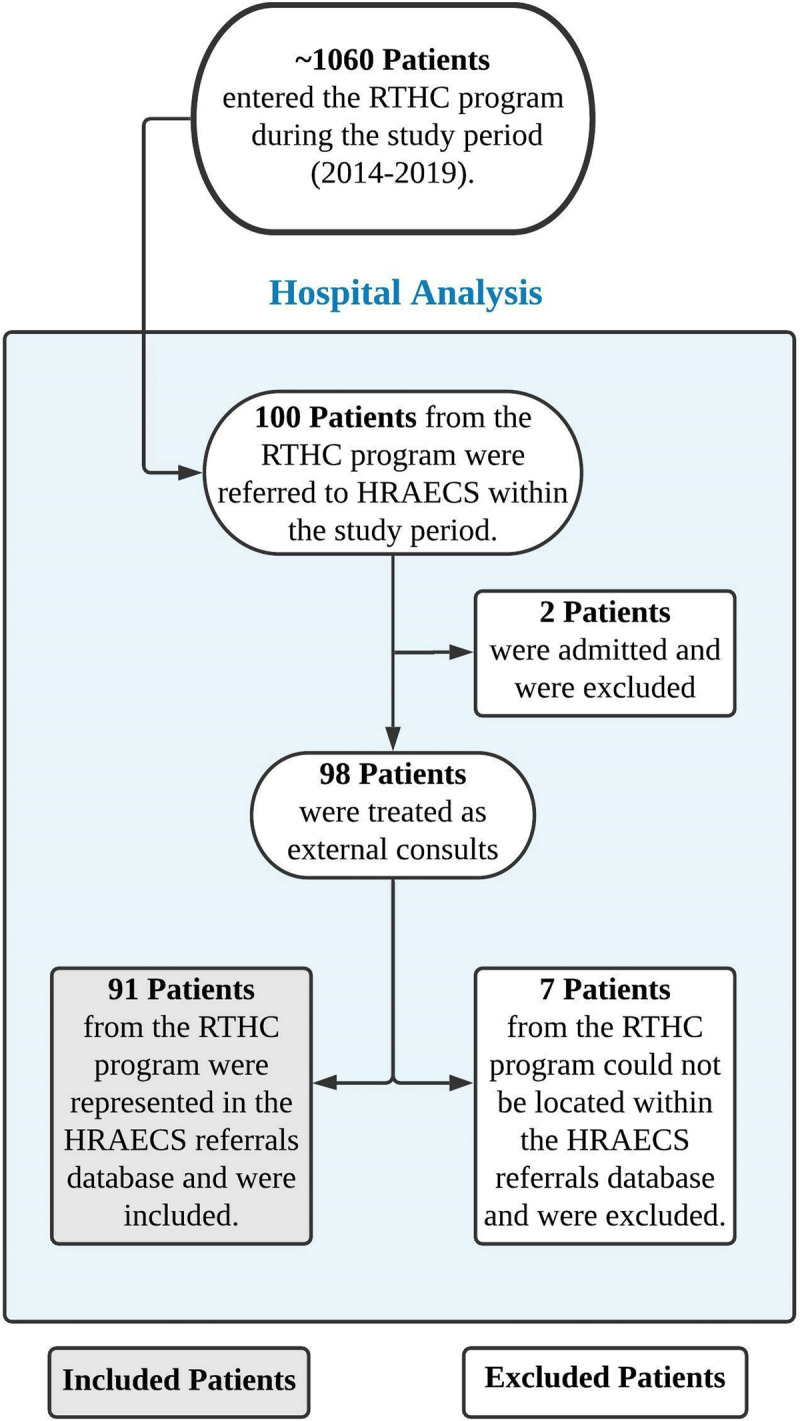
Inclusion criteria and patient counts. HRAECS = Hospital Regional de la Alta Especialidad Ciudad Salud.

The current study was a retrospective analysis examining the impact of the RTHC program on process outcomes of the referral system to tertiary care. Data collection began using internal databases maintained by the RTHC program. All patients enrolled in the RTHC program from calendar years 2014 through 2019 and who were identified for referral to Hospital Ciudad Salud were considered eligible for the study (100 patients). For these 100 patients, records were matched with the referral databases at Hospital Ciudad Salud. Seven patients’ records could not be found at Hospital Ciudad Salud and were excluded. These patients’ referrals were almost certainly never submitted to Hospital Ciudad Salud, as any received referral (regardless of acceptance or denial) is recorded. These patients likely withdrew from the RTHC program, sought care elsewhere, or passed away before the referral was submitted. Given that exclusion was not associated with the outcome, the research team felt that these numbers were acceptable and proceeded with the analysis. Of the remaining 93 patients, 2 were admitted to the hospital and received inpatient treatment, and the remaining patients were treated as outpatients. Due to the significant differences in the referral process for admitted patients compared to those receiving outpatient treatment, these two patients were also excluded, leaving a final study cohort of 91 patients. Inclusion criteria and counts are summarized in ***Figure 2***.

Databases from Hospital Ciudad Salud were the source of all variables used in the analysis. First, yearly referral databases were combined and cleaned using Tableau Prep (Version 2021.1). Variables extracted directly included patient name, sex, and age, as well as referral result, date received, date replied, and appointment date if applicable. Specialty referred to was also collected and grouped into clinically relevant groupings to facilitate patient matching. Patient location was extracted from a manually entered free text field and grouped into official municipalities. The level of the referring healthcare facility was extracted using naming guidelines set forth by the public sector [23], using published information from the respective facility, and through speaking with local clinicians.

The dataset was then appended with two additional sources of information. First, patients who met inclusion criteria and were enrolled in the RTHC were marked, using manual comparison of patient name, age, and referral date to internal Compañeros en Salud records. At this point patients were matched. For the 91 RTHC patients who met inclusion criteria, and for the matched cohort, additional data points were collected from the Hospital Ciudad Salud patient registration database. Every patient who attends an appointment at Hospital Ciudad Salud is entered into this database after their appointment and is assigned a unique Patient Record Number. This number is only assigned to patients after they attend their first appointment with a physician. If a patient referral is accepted and an appointment date is assigned, but the patient never attends the appointment for any reason, they will not have a Patient Record Number. Therefore, the presence of a Patient Record Number allowed the research team to see which patients physically attended their appointments. The study dataset for included and matched patients was appended with whether the patient had a Patient Record Number, and the date of registration. This was done like above with manual review. All manual data review was confirmed by a second researcher.

### Data Analysis

Prior to matching, the included patient dataset was further processed to assign each patient in the database a unique ID, and to group patients with multiple referrals to the same unique ID. Twenty-eight entries in the Hospital Ciudad Salud dataset were excluded due to the presence of null values (0.5% of total. entries, all from the potential control cohort). Next, power calculations were done in R (Version: 3.6.0) and 2:1 patient matching was selected to improve statistical power within the limits of the time and resources of the research team. Patient matching of RTHC program patients with non-RTHC controls was done using the “Matchit” package in R. Controls were defined as patients referred to Hospital Ciudad Salud as external consults who were unaffiliated with the RTHC program. Optimal pair matching was performed matching two controls to each patient in the RTHC program, balancing for the covariates which could affect a patient’s success in the referral process. Covariates were selected based on discussion with local clinicians and data availability, and included patient age, sex, specialty referred to, level of referring hospital, and the patient’s municipality. As Hospital Ciudad Salud is a public sector hospital, all patients in the study do not have a social insurance scheme or private insurance and are relying exclusively on their public sector benefits. Optimal pair matching was selected given slightly improved balance compared to nearest neighbor matching. Characteristics of the control group before matching, the matched controls, and the treatment group are compared below in ***Table 1***. Outcomes analysis was done in R using the “epitools” package.

**Table 1:** Summary of matching results.


COVARIATES	ALL ELIGIBLE CONTROLS (N = 4470)	MATCHED	

		CONTROL (N = 182)	TREATMENT (N = 91)

**Specialty Referred To**			

Oncology	16.2%	33.5%	39.6%

Internal Medicine	29.2%	21.4%	20.9%

Other Surgical Subspecialties	11.8%	17.0%	16.5%

General Surgery	8.9%	7.7%	7.7%

Neurosurgery	6.0%	10.4%	4.4%

Urology	10.1%	3.3%	4.4%

Gynecology	4.7%	1.7%	2.2%

Neurology	4.6%	2.8%	2.2%

Hematology	0.0%	0.0%	1.1%

Trauma	6.7%	2.2%	1.1%

**Sex**			

Female	58.7%	47.8%	51.7%

Male	41.3%	52.2%	48.4%

**Age**			

Mean Age in Years (Range)	49.9	51.9 (16–89)	51.4 (20–86)

**Level of Referring Hospital**			

Primary	36.1%	41.2%	47.3%

Secondary	49.7%	24.2%	13.2%

Tertiary	1.4%	1.1%	1.1%

Unknown	12.8%	33.5%	38.5%

**Municipality**			

Ángel Albino Corzo	1.2%	24.7%	35.2%

Siltepec	7.8%	41.8%	26.4%

La Concordia	1.3%	18.1%	17.6%

Montecristo de Guerrero	0.4%	8.8%	12.1%

Capitán Luis Ángel Vidal	0.0%	0.6%	1.1%

Chiapa de Corzo	1.6%	1.1%	1.1%

Chicomuselo	2.8%	0.0%	1.1%

Escuintla	9.1%	0.6%	1.1%

Motozintla	24.9%	1.7%	1.1%

Pijijiapan	33.0%	2.8%	1.1%

Tuxtla Gutiérrez	16.3%	0.0%	1.1%

Villa Corzo	1.5%	0.0%	1.1%


### Outcomes

The primary outcomes analyzed were if the patient had any referral accepted (regardless of number of iterations), and if the patient had attended any appointment. The secondary outcomes were the program’s impact on the frequency of acceptance of the first referral, and the program’s direct impact on the attendance rate of appointments for patients with an accepted referral. The approval of the first referral was analyzed independently as a secondary objective to help elicit where in the referral process the RTHC was having an impact, allowing independent analysis of the impact of the quality of the initial referral and the effects of following up, modifying, and resubmitting rejected referrals. Attendance rate for patients who had an accepted referral was analyzed independently to delineate outcomes due to support at different stages of the referral process. Specifically, this separates the effects of the RTHC program on referral acceptance from the effects on physical attendance of medical appointments. Lastly, descriptive counts were included for patients who did not have their initial referral approved, to help understand where in the referral process the RTHC program was having an impact.

## Results

Ninety out of 91 RTHC patients had an accepted referral (99%), compared to 149 of the 182 patients in the matched control group (82%). Odds of any accepted referral were significantly higher for patients in the RTHC program compared to the control group (OR 17.42, 95% CI 3.68 to 414.16). When reviewing patient registration records, 80 of the 91 RTHC patients had attended an appointment during the study period (88%), compared to 103 of the 182 patients in the matched control group (57%). Odds of attending an appointment were significantly higher for RTHC patients compared to the control group (OR 5.49, 95% CI 2.93 to 11.60).

In the RTHC group, 83 of 91 patients had their first referral accepted (91%), compared to 143 of 182 patients in the matched control group (79%). Odds of first-time acceptance were significantly higher for patients in the RTHC program compared to the control group (OR 2.78, 95% CI 1.29 to 6.73). Subset analysis of accepted patients elicited the differences in appointment attendance rate, with 80 of the 90 (89%) accepted RTHC patients attending an appointment, compared to 100 of 149 (67%) accepted patients in the matched control group (OR 3.86, 95% CI 1.90 to 8.57).

When looking at patient counts to elicit where the RTHC program is having an impact, in addition to the above outcomes it is worth highlighting that of the 8 patients in the RTHC program who had their referral initially rejected, all 8 reapplied, with 7 of the 8 having their referral accepted after iterations (100% and 88% respectively). In comparison, of the 39 patients who were initially rejected in the matched cohort, only 7 reapplied, with 6 of the 7 being approved after iteration. (18% and 86% respectively). Outcomes are summarized in ***Table 2*** below.

**Table 2:** Summary of study outcomes.


OUTCOME	RTHC GROUP (TREATMENT)	MATCHED GROUP (CONTROL)	ODDS RATIO	95% CI

**1.1:** Any referral accepted	99% (90/91)	82% (149/182)	17.42	3.68 – 414.16

**1.2:** Any appointment attended	88% (80/91)	57% (103/182)	5.49	2.93 – 11.60

**2.1:** First-time referral accepted	91% (83/91)	79% (143/182)	2.78	1.29 – 6.73

**2.2:** Attendance rate for accepted patients	89% (80/90)	67% (100/149)	3.86	1.90 – 8.57


## Discussion

This study is the first outcomes analysis of the RTHC program, which represents a novel social work model which could be adapted to improve specialty care access elsewhere in rural Mexico, in Low-to-Middle-Income Countries (LMICs), or for vulnerable populations in High Income Countries (HICs). The results of the study are highly positive. This reflects both the magnitude of barriers patients face when seeking specialty care in the region, and the success of the RTHC program at helping patients overcome these barriers. It is important to interpret these results within the context of the referral process to tertiary care in Chiapas and the various points at which patients face barriers to access.

As illustrated in the diagram, the outcomes illuminate the program’s impact on different steps of the referral process. The RTHC program facilitates initializing the referral process, provides guidance to physician’s submitting referral forms, and coordinates and provides economic support for diagnostic studies. While not individually assessed, the collective of these interventions can be seen in the primary outcome of having any referral accepted, as patients had greater odds of having a referral accepted if they were enrolled in the RTHC program. Similarly, the secondary outcome of first-time referral acceptance also demonstrates significantly greater odds of acceptance for patients in the intervention group. Both outcomes speak to the increased quality of the referrals being submitted. Likely mechanisms behind these improved outcomes are a synergy of the interventions just discussed, facilitated by a dedicated interdisciplinary referral team through the RTHC program. Economic support and coordination with private laboratories ensure that the proper laboratory results and imaging studies accompany the referral form. Finally, institutional knowledge of hospitals in the region through the social work team facilitates referrals to the correct hospital with the required documentation.

The next primary outcome, attendance of any appointment, demonstrates that the effects observed on referral acceptance rate led to the significantly increased appointment attendance for the RTHC program compared to the matched control group. Of all the outcomes studied, this most directly demonstrates that the RTHC program is having its intended clinical impact—increasing patient access to specialty care.

Finally, appointment attendance rate for the subset of patients who had a referral accepted elicits an important distinction in the results. By demonstrating significantly increased odds of attending an appointment for RTHC patients with an accepted referral when compared to patients in the matched control group with an accepted referral, this outcome shows that the RTHC program is independently effective at increasing the odds that a patient attends their appointment. In other words, this outcome demonstrates that the impact of increased appointment attendance is not only due to an increase in referral acceptance, but also due to a direct impact on an increase in patient appointment attendance rate. This is likely due to the economic and patient navigation support provided by the RTHC program to help patients overcome the barriers outlined prior in the introduction. Additionally, accompaniment of patients throughout the process likely contributed to the increased appointment attendance rate.

To further clarify the impact of the program on patients initially rejected, further descriptive patient counts are highlighted. Most notably, there was a difference between the treatment and control group in the number of rejected patients who reapplied. While 100% of rejected referrals in the RTHC group later reapplied, only 18% rejected matched controls ever reapplied, suggesting that one part of the observed benefit of the RTHC program comes from successfully following up with initially rejected referrals, completing the requested changes, and successfully reapplying. Given the logistical complexity of the referral process, it is not surprising that a dedicated referral team as utilized in the RTHC program does improve follow up, correction, and resubmission for rejected patients. In conclusion, the results speak to the magnitude of the barriers faced by patients in the region, as shown by only 57% of referred patients in the control group ever attended an appointment, and the success of the RTHC program in helping patients overcome these barriers. This success is demonstrated in all outcomes analyzed.

This study looks at a comprehensive referral support program, for which there is no equivalent documented in the literature. There is no comparable program elsewhere in Mexico. Even when looking for a comparison internationally, comparable programs usually address a specific unmet social determinant of health need. In contrast, the RTHC program is comprehensive, addressing many barriers to care which arise from social determinants through social work, patient navigation, financial support, and accompaniment. One example of an existing program is Medicaid transportation vans in the United States. In North Carolina, Project Access works to address disparities in access to specialty care; however, Project Access directly funds or coordinates the donations of specialty care for patients without insurance [24]. While Project Access serves patients who neither qualify for government insurance nor make enough money to purchase private insurance, the RTHC program works to facilitate access to the public sector and fill the gaps in services when necessary. While the RTHC program is unique, the results are consistent with prior work that has shown the success of addressing specific individual barriers to access. For example, Medicaid transportation services when implemented were demonstrated to increase access and decrease overall expenditures due to a reduction in the use of emergency services and hospitalizations [25]. While health system cost-savings were not included in this study, it is important to highlight the precedent that increasing access can reduce overall healthcare costs. Another powerful example of the effects of increasing access is shown in a recent study of trauma patients in the United States. While racial disparities in trauma care between Black and White patients have been thoroughly documented by systemic review and meta-analysis [26], this large-scale study found that among patients with TRICARE military health coverage, racial disparities were eliminated, suggesting that an equal-access healthcare system could help remedy existing health disparities in trauma care [27].

One limitation of this study is that the location covariate balanced in the matched control group was only able to be matched to the level of municipalities, which are a geographic level roughly equivalent to counties in the United States. In the study region municipalities center around a town which serves as the municipal center. While RTHC patients come from small rural communities in the mountains, the control group is biased towards larger towns and municipal centers due to the population distribution favoring those areas. Given the large relative increase in development and proximity to infrastructure and health services in municipal centers, the control group likely does not face the same magnitude of barriers as the treatment group. The effect of this limitation is that the treatment effect of the RTHC is likely underestimated. The current study was also limited to one tertiary care hospital, and future research is needed to demonstrate to what level the results are transferable to other hospitals.

Additionally, a large portion of RTHC resources, both in staff and program expenses, is spent supporting patients throughout their treatment. This includes continued social work support, case management, and direct coverage of medicines and surgical supplies unavailable through the public sector but necessary for patients’ continued treatment. The impact of this continued support was not able to be measured within the scope of this study and should be a priority for future research. Future research should also further clarify the effects of the different modalities of support offered by the RTHC program. When comparing the results in ***Table 2*** with the program interventions in ***Figure 1***, this study suggests that the positive impacts on the outcomes are due to both patient navigation support (which is the primary mode of support prior to referral acceptance) and social work and financial support (which are the primary modes of support after referral acceptance). However, it is the belief of the authors and team which operates the RTHC program that a large part of the program’s success is due to the synergy of patient navigation support, social work, and financial support. This comprehensive approach, grounded by assessments of individual patient needs, drives the success of the program. In contrast, if the individual interventions of the program were administered in isolation, their impact would be hindered by unmet needs in other areas. Future research should also explore the effects of age, diagnosis group, and other variables on the impact of the RTHC program.

Another suggestion for future research is the effectiveness of utilizing telemedicine services to increase access to specialty care in the region. A recent global review highlights the promising results of using telemedicine for increasing specialty care access; however the only Latin American country included in the review was Brazil [28]. Together, this suggests the need to pilot and evaluate the use of telemedicine to increase specialty care access in Latin America, which could be incorporated into the RTHC program in the future. Lastly, future research should evaluate the use of a similar referral program in different contexts, including the urban poor, other LMICs, and HICs, as barriers to specialty care are not limited to the study setting. For example, difficulty accessing specialty care is reported among patients treated in community health centers throughout the United States of America, especially among uninsured patients [29]. Research should focus on evaluating the impact of the investment in services increasing access to specialty care, as the current paradigm places the burden of costs, logistics, and navigation of increasingly complex health systems, on patients and their support networks.

## Conclusion

A small but growing body of evidence suggests the RTHC program represents an effective, context-specific model for increasing access to specialty care. Given the program’s uniqueness and demonstrated effectiveness, the results of this study advocate for its continued funding, expansion, and further research. It is possible that the program could be adapted to successfully meet patient needs elsewhere in Mexico, in other LMICs, or for vulnerable populations in HICs.

## Verification Statement

All authors had access to the data and a role in writing the manuscript.
